# XcisClique: analysis of regulatory bicliques

**DOI:** 10.1186/1471-2105-7-218

**Published:** 2006-04-21

**Authors:** Amrita Pati, Cecilia Vasquez-Robinet, Lenwood S Heath, Ruth Grene, TM Murali

**Affiliations:** 1 Department of Computer Science, Virginia Polytechnic Institute and State University, Blacksburg, VA 24061, USA; 2 Department of Plant Pathology, Physiology, and Weed Science, Virginia Polytechnic Institute and State University, Blacksburg, VA 24061, USA

## Abstract

**Background:**

Modeling of *cis*-elements or regulatory motifs in promoter (upstream) regions of genes is a challenging computational problem. In this work, set of regulatory motifs simultaneously present in the promoters of a set of genes is modeled as a biclique in a suitably defined bipartite graph. A biologically meaningful co-occurrence of multiple *cis*-elements in a gene promoter is assessed by the combined analysis of genomic and gene expression data. Greater statistical significance is associated with a set of genes that shares a common set of regulatory motifs, while simultaneously exhibiting highly correlated gene expression under given experimental conditions.

**Methods:**

XcisClique, the system developed in this work, is a comprehensive infrastructure that associates annotated genome and gene expression data, models known *cis*-elements as regular expressions, identifies maximal bicliques in a bipartite gene-motif graph; and ranks bicliques based on their computed statistical significance. Significance is a function of the probability of occurrence of those motifs in a biclique (a hypergeometric distribution), and on the new sum of absolute values statistic (SAV) that uses Spearman correlations of gene expression vectors. SAV is a statistic well-suited for this purpose as described in the discussion.

**Results:**

XcisClique identifies new motif and gene combinations that might indicate as yet unidentified involvement of sets of genes in biological functions and processes. It currently supports *Arabidopsis thaliana *and can be adapted to other organisms, assuming the existence of annotated genomic sequences, suitable gene expression data, and identified regulatory motifs. A subset of Xcis Clique functionalities, including the motif visualization component MotifSee, source code, and supplementary material are available at .

## Background

Gene regulation is an intricate, dynamic phenomenon essential for all biological functions including cell metabolism, development, and response to environmental stress and pathogen attack. Primary actors include transcription factors (TFs), which recognize and bind to specific DNA sequences in gene promoters. These DNA sequences are known variously as *cis*-elements, transcription factor binding sites (TFBSs), or regulatory motifs. Here, these terms are used interchangeably.

The binding strength of a TF for a given *cis*-element depends on the precise DNA sequence, while each *cis*-element has binding affinity for a particular subset of all TFs. The details determining differential receptivity of a transcription factor for different sequences is not yet known, but sequence specificity and conformational changes are likely to be involved [1]. Regulation of transcription depends on the binding of one or more TFs to corresponding *cis*-elements in a gene promoter, which may initiate, terminate, enhance, or repress transcription. TFs are often large proteins or protein-complexes, and this imposes geometric and spatial constraints on the separation between and arrangement of *cis*-elements [2]. The rate of transcription, and hence, gene expression depends on the combination of TFs currently bound to the regulatory regions of genes [3,4]. Often, the sequence distance from the TATA box to the *cis*-element binding a TF influences the amount of control that the TF has on gene transcription [5]. In summary, transcriptional regulation of a gene depends on a number of factors, including these: the *cis*-elements present in the gene promoter; the distances between *cis*-elements; the order of *cis*-elements; and the distance from a *cis*-element to the transcription start site.

In the past decade, a number of computational tools have been developed to analyze the promoters of various organisms. These tools fall into three broad categories. Tools in the first category discover or identify gene promoters from nucleotide sequences [3,6]. Tools in the second category predict putative *cis*-elements in the promoters of a family of genes using pattern discovery and pattern matching techniques. [7-11], and [12], describe and compare such tools that use both enumerative and probabilistic approaches. Tools in the third category model and analyze the presence of combinations of *cis*-elements in gene promoters and the effect of these combinations on the regulation of transcription. Examples of tools in this category are found in [4,13,14], and [15].

The XcisClique system has been developed to incorporate genomic and *cis*-element data for *Arabidopsis thaliana *(AT). Pilpel, et al. [4] identify regulatory networks in yeast by building a database of known and putative yeast TFBSs and identifying synergistic motif combinations based on the expression coherence score of each gene set having a pair of motifs. Their motif-association maps are highly connected, indicating that transcription factors work in combinations to render different expression patterns and that motif co-occurrence is essential for transcriptional regulation. However, they use position weight matrices (PWMs) for modeling binding sites; PWMs for *Arabidopsis thaliana *are rare with TRANSFAC containing PWMs for just 10 AT binding sites. Kato, et al. [14] integrate chromatin-immunoprecipitation (ChIP) data available for yeast with combinatorial motif analysis to identify over-represented motif combinations. Genome-wide ChIP data are rarely available for other organisms and are not available for AT. Chiang, et al. [13] identify regulatory templates consisting of pairs of hexamers identified in yeast genomes as conserved in co-occurrence and spatial separation. A common drawback of the probabilistic methods such as those used in [13] and [15] is that they consider *n*-mers only (typically hexamers). These methods discover regulatory templates and not the actual motifs. Furthermore, since, in most cases, the specific *cis*-element regions for each of the members of a given transcription factor family have not yet been determined, what is currently available is a consensus sequence serving as a motif rather than a specific sequence. TFBSs in AT vary widely in length; for instance, the heat shock element in *Arabidopsis thaliana (AT) *is 13 nucleotides long, while the ACGTATERD1 (PLACE identifier) motif is 4 nucleotides long.

Integrating motif discovery into identification of motif combinations leads to the discovery of a very large number of combinations (exponential) of regulatory templates. Also, probabilistic approaches require that the predictive models be trained in an organism-specific manner. The optimal model parameters to use are dependent upon the organism, the tissue type, the regulatory process, and the particular TFBSs. Most probabilistic models use yeast as their model organism. Yeast is a much more widely studied organism as compared with *Arabidopsis *and data for yeast is available on a much larger scale. Because of the above reasons, XcisClique excludes motif discovery from the system and uses only known motifs to identify over-represented motif combinations. A preliminary analysis of spatial conservation of motif-pairs in AT promoters was done to determine the inclusion of spatial conservation of regulatory elements in combinatorial analysis. We did not find any patterns that suggest conservation of spacing between pairs of *cis*-elements in AT. This may be due to limitations of the current known *cis*-elements for AT. So, XcisClique uses the presence of combinations of *cis*-elements to derive regulatory bicliques. *cis*-regulatory motifs are properly represented as strings over the alphabet {*A*, *B*, *C*, *D G*, *H*, *K*, *N*, *M*, *R*, *S*,*T*, *V*, *W*, *Y*}, the IUPAC recommended alphabet for bases in nucleic acid sequences IN the case of AT, many of these motifs are consensus sequences. [16]. A *motifset *is any set of regulatory motifs. The presence of the members of a motifset in the promoters of two or more genes have biological significance in that those motifs may participate in the co-regulation of those genes. The number of distinct motifsets present in the promoters of genes in any genome is quite large, typically exponential in the number of motifs considered. Hence, exhaustively analyzing all motifsets is too expensive computationally.

More naturally, a biologist starts with a *geneset*, a set of genes of interest. Typically, a geneset is small and consists of genes that are co-regulated under some treatments, and the biologist wishes to identify motifsets common to some of the genes that have biological significance. The number of motifsets identified as co-occurring in subsets of the geneset of interest is still, typically, quite large. The computational setting is best expressed as a bipartite graph with vertices that are either for example, *Arabidopsis *genes or motifs and with edges that connect a gene and a motif if the motif occurs in the promoter of the gene. Then, each subgraph of interest is a *regulatory biclique*, a geneset and a motifset for which every gene in the geneset is adjacent to every motif in the motifset. The statistical significance of a motifset can be assessed using the hypergeometric distribution applied to the occurrence of the motifset in the entire *Arabidopsis *genome. The statistical significance of a geneset *(vis-à-vis *co-expression) can be assessed using correlation of gene expression from microarray experiments. The statistical significance of a biclique is then a combination of the significance for the geneset and the motifset. Biclique significance allows for the identification of the most important motifsets in a particular biological context. For example, some AT *cis*-elements, such as those related to water stress, are present in the promoters of a large fraction (> 89%) of genes in the genome. Consequently, water stress elements appear in many significant bicliques and their presence contributes little to the statistical significance of a biclique. Hence, the biclique obtained by deleting water stress elements remains statistically significant.

Here, we present the XcisClique system, which integrates the *Arabidopsis *genome with gene expression data to identify statistically significant regulatory bicliques for genesets of interest [17]. In particular, XcisClique uses the Apriori algorithm [18,19] to identify *maximal regulatory bicliques*, which are bicliques that cannot be made larger by the addition of any gene in the geneset of interest or by the addition of any motif in the known set of regulatory motifs. Due to the lack of reliable tools to predict *Arabidopsis *regulatory motifs and to reduce the search space to include only known regulatory motifs, XcisClique has no motif discovery component. Rather, XcisClique employs known motifs from the PLACE database [20]. With XcisClique, it is computationally feasible to identify maximal bicliques and to assess their statistical significance for genesets consisting of a few hundred genes and our current set of several hundred regulatory motifs.

## Implementation

### Annotated genome data

Using Perl scripts and the Entrez Programming Utilities [21], we populated a PostgreSQL database of *Arabidopsis *genes, proteins, and promoters.

### Microarray expression data

Expression data for the AT transcriptome was retrieved from NASC arrays in the Nottingham database ([22]). The slides are Affymetrix ATH1 AT Genome Arrays having 22, 814 genes. These data were generated as part of the AtGenExpress project funded by Das von der DFG geforderte AFGN (Arabidopsis Functional Genomics), which aims to provide the AT community with access to a large set of Affymetrix microarray data. This project generated expression data from 80 biologically different samples and analyzed the data using the Affymetrix Microarray Analysis Suite 5.0 with the Affymetrix MAS 5.0 Scaling Protocol. We selected 272 slides organized as follows. There are 9 abiotic stress experiments, with these stress treatments: Salt, Drought, Genotoxic, Oxidative, UV-B, Wounding, Heat, Cold, and Osmotic. Expression data for each of these is available over a series of time points (some of 0.25 h, 0.5 h, 1 h, 3 h, 6 h, 12 h, 24 h) with two biological replicates per time-point. Control slides also exist for each of these time points. We retained the following five time points, which are common to all 9 treatments: 0.5 h, 1 h, 3 h, 6 h, and 12 h. All expression data are intensity values. Half (136) of the 272 slides contain experiments involving shoots and, the other half (136) slides contain experiments involving roots.

### cis-element data

PLACE, a database of plant *cis*-acting regulatory elements [20], is our primary source for *cis*-regulatory element data. These have been compiled from previously published reports and cover vascular plants only. Their variations in other genes or in plant species are also reported along with literature references. XcisClique uses the subset of AT *cis*-elements present in the POPS database. Additional analysis-specific AT motifs have also been curated from various sources in literature. The POPS database contains 276 *Arabidopsis *motifs in all; 9 of these have been curated from [23], 47 of these are heat shock elements.

### Graph theoretic setting

For a geneset *G *and a motifset *P *(given as regular expressions), the *occurrence graph *O
 MathType@MTEF@5@5@+=feaafiart1ev1aaatCvAUfKttLearuWrP9MDH5MBPbIqV92AaeXatLxBI9gBamrtHrhAL1wy0L2yHvtyaeHbnfgDOvwBHrxAJfwnaebbnrfifHhDYfgasaacH8akY=wiFfYdH8Gipec8Eeeu0xXdbba9frFj0=OqFfea0dXdd9vqai=hGuQ8kuc9pgc9s8qqaq=dirpe0xb9q8qiLsFr0=vr0=vr0dc8meaabaqaciaacaGaaeqabaWaaeGaeaaakeaaimaacqWFoe=taaa@383D@ = (G, P, E) of *G *and *P *is the bipartite graph that has *g ∈ G *adjacent to *p ∈ P *if *p *occurs in the promoter of *g *.A *cis*-element is modeled as a Perl regular expression by manually consolidating all its available forms from PLACE and/or literature, and manually synthesizing a regular expression that matches all the forms. Available forms of *cis*-elements were taken from PLACE and literature. For instance, the metal responsive element (MRE) was specified to have a consensus sequence of TGCRCNC in PLACE and sequences TGCGCAAC and TGCAGAC in literature. So the Perl regular expression for an MRE is (TGCRCNC)|(TGCGCAAC)|(TGCAGAC). The database has 9 *cis*-elements whose regular expressions have been synthesized using the above process. Its location in the promoters of genes is determined by exact pattern matching. A *biclique *for *G *and *P *is a complete bipartite graph in O
 MathType@MTEF@5@5@+=feaafiart1ev1aaatCvAUfKttLearuWrP9MDH5MBPbIqV92AaeXatLxBI9gBamrtHrhAL1wy0L2yHvtyaeHbnfgDOvwBHrxAJfwnaebbnrfifHhDYfgasaacH8akY=wiFfYdH8Gipec8Eeeu0xXdbba9frFj0=OqFfea0dXdd9vqai=hGuQ8kuc9pgc9s8qqaq=dirpe0xb9q8qiLsFr0=vr0=vr0dc8meaabaqaciaacaGaaeqabaWaaeGaeaaakeaaimaacqWFoe=taaa@383D@, which is a geneset *G' *⊂ *G *and a motifset *P' ⊂ P *such that every gene in *G' *is adjacent to every pattern in *P' *.We write ⟨*G*', *P' *⟩ for the biclique. Biclique ⟨*G'*, *P' *⟩ is *maximal *if there is no gene *g ∈ G – G' *such that ⟨*G' *∪ {*g*}, *P'*⟩ is a biclique and there is no pattern *p ∈ P – P' *such that ⟨*G'*, *P' *∪{*p'*}⟩ is a biclique.

The following is an example of a biclique from an analysis done by XcisClique. The input set of genes is set of 17 genes involved in stress, pathogenicity, and secondary metabolism in AT [24].

Expression data for 15 of the 17 genes is available in the POPS database. Promoters of length 1200 for the input geneset were scanned for the set of all AT *cis*-elements. Expression data were correlated over a set of 7 treatments (Cold, Heat, Drought, Osmotic, Oxidative, Salt, UVB) in shoots. The 32^*nd *^biclique *I*_32 _identified by the Apriori algorithm has a *p*-value of 3.955 × 10^-03 ^from sequence analysis and a *p-*value of 1.236 × 10^-02 ^from expression data analysis.

**Figure 1:**
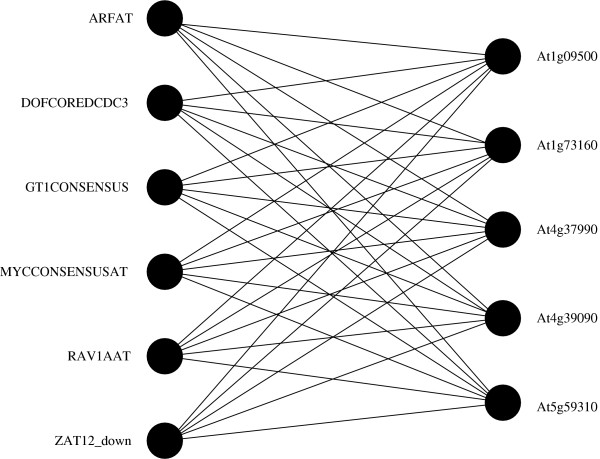
Graphical representation of a Biclique. The vertices of a biclique can be partitioned into two sets *S *and *T *such that no two vertices within a set are adjacent and every vertex in *S*is connected to every vertex in *T *and vice-versa. In this case, *S *is the set of genes and *T *is the set of motifs.

*I*_32 _= ⟨ *G*_32_, *M*_32 _⟩ where *G*_32 _consists of genes {Atlg09500, Atlg73160, At4g37990, At4g39090, At5g59310} and *M*_32 _consists of motifs {ARFAT, DPBFCOREDCDC3, GT1CONSENSUS, MYCCONSENSUSAT, RAV1AAT, ZAT12-down}. Figure 1 illustrates the biclique that models ***I***_32_.

### XcisClique overview

**Figure 2:**
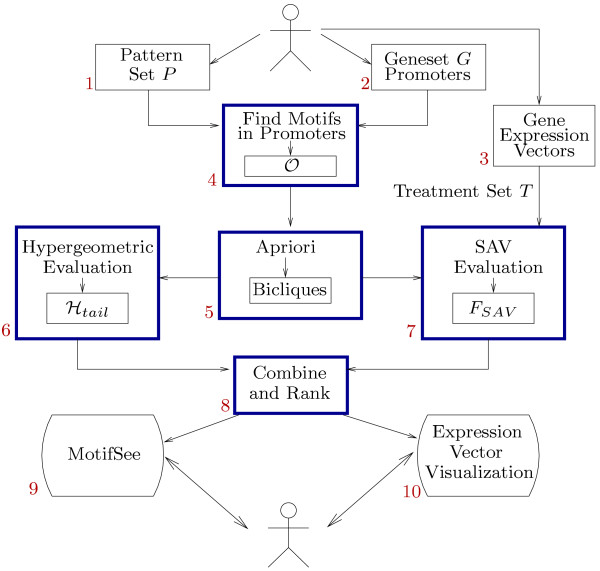
XcisClique Schematic. Contents of thickly outlined boxes indicate processing. Contents of thinly outlined boxes describe data. 1. Input geneset *G*, 2. Input set of patterns *P*, 3. Input set of treatments T, 4. Find matches of *P *on promoters of *G *to get occurrence graph O
 MathType@MTEF@5@5@+=feaafiart1ev1aaatCvAUfKttLearuWrP9MDH5MBPbIqV92AaeXatLxBI9gBamrtHrhAL1wy0L2yHvtyaeHbnfgDOvwBHrxAJfwnaebbnrfifHhDYfgasaacH8akY=wiFfYdH8Gipec8Eeeu0xXdbba9frFj0=OqFfea0dXdd9vqai=hGuQ8kuc9pgc9s8qqaq=dirpe0xb9q8qiLsFr0=vr0=vr0dc8meaabaqaciaacaGaaeqabaWaaeGaeaaakeaaimaacqWFoe=taaa@383D@, 5. Feed O
 MathType@MTEF@5@5@+=feaafiart1ev1aaatCvAUfKttLearuWrP9MDH5MBPbIqV92AaeXatLxBI9gBamrtHrhAL1wy0L2yHvtyaeHbnfgDOvwBHrxAJfwnaebbnrfifHhDYfgasaacH8akY=wiFfYdH8Gipec8Eeeu0xXdbba9frFj0=OqFfea0dXdd9vqai=hGuQ8kuc9pgc9s8qqaq=dirpe0xb9q8qiLsFr0=vr0=vr0dc8meaabaqaciaacaGaaeqabaWaaeGaeaaakeaaimaacqWFoe=taaa@383D@ to Apriori to identify bicliques, 6. Evaluate bicliques with respect to statistical over-representation of patterns to get ℋ
 MathType@MTEF@5@5@+=feaafiart1ev1aaatCvAUfKttLearuWrP9MDH5MBPbIqV92AaeXatLxBI9gBamrtHrhAL1wy0L2yHvtyaeHbnfgDOvwBHrxAJfwnaebbnrfifHhDYfgasaacH8akY=wiFfYdH8Gipec8Eeeu0xXdbba9frFj0=OqFfea0dXdd9vqai=hGuQ8kuc9pgc9s8qqaq=dirpe0xb9q8qiLsFr0=vr0=vr0dc8meaabaqaciaacaGaaeqabaWaaeGaeaaakeaaimaacqWFlecsaaa@3763@_*tail*_, 7. Evaluate bicliques over *T *to get *F*_*SAV*_, 8. Combine ℋ
 MathType@MTEF@5@5@+=feaafiart1ev1aaatCvAUfKttLearuWrP9MDH5MBPbIqV92AaeXatLxBI9gBamrtHrhAL1wy0L2yHvtyaeHbnfgDOvwBHrxAJfwnaebbnrfifHhDYfgasaacH8akY=wiFfYdH8Gipec8Eeeu0xXdbba9frFj0=OqFfea0dXdd9vqai=hGuQ8kuc9pgc9s8qqaq=dirpe0xb9q8qiLsFr0=vr0=vr0dc8meaabaqaciaacaGaaeqabaWaaeGaeaaakeaaimaacqWFlecsaaa@3763@_*tail *_and *F*_*SAV *_for each biclique and rank bicliques, 9. Visualize arrangement of *cis*-elements on promoters, biclique-wise, 10. Visualize gene expression vectors biclique-wise.

Figure 2 depicts the process flow in XcisClique. XcisClique is an integrated suite of programs in Perl, Matlab, and C++, much of which is directly accessible through its web site. There are three kinds of user input: a set of AGI numbers *G*, corresponding to a geneset of interest; the set *P *of patterns, corresponding to *cis*-elements of interest, typically selected from the database of regulatory elements in XcisClique; and the treatment set *T *of interest, from which expression vectors are correlated.

The output from XcisClique feeds into the visualization tool MotifSee. This is a web-based tool, implemented in PHP, that accepts input tuples in the format ⟨ Gene, Motif, Sequence, Start_Position, End_Position, TATA_start, TATA_end ⟩. Besides visualizing *cis*-elements exactly as they occur on the promoters, this tool allows viewing subsets of genes and *cis*-elements as well as subsequences of promoters. XcisClique also has a viewer for gene expression vectors integrated into it to visualize expression patterns of genes in a biclique. The web site for XcisClique is hosted at [25].

### Gene expression vectors

XcisClique processed gene expression data for 22,814 genes to extract tissue-specific time series data vectors. There are 9 treatments and 5 time points per treatment in the POPS database. Let *e*_*g*,*k*,*t *_be the ratio of treated and control expression for gene *g*, a particular treatment *k*, and time *t *. While XcisClique can process any user-specified subset of the 9 treatments, for convenience of exposition, we assume that all 9 treatments are used. Let *g *be any gene. Define the expression vector for *v *to be the 45-component vector

*v*_*g *_= (*e*_*g*,1,1_, *e*_*g*1,2_, *e*_*g*,1,3_, *e*_*g*,1,4_, *e*_*g*,1,5_, *e*_*g*,2,1_,...,*e*_*g*,9,4_, *e*_*g*,9,5_).

(More generally, if *z *treatments are used, then *v*_*g *_is a 5*z*-component vector.) XcisClique uses correlation between gene expression vectors to assess potential co-regulation of genes. XcisClique computes the Spearman correlation coefficient ρ*(v*_*g*1_, *v*_*g*2_*) *each gene *g*_1 _among the 22,814 genes and between each gene *g*_2 _in the geneset of interest. The distribution of ρ-values for the correlation of a gene with all other genes of the genome is approximately normal as illustrated in Supplementary Figure 1 [See Additional file 1]. This distribution can be used to compute an estimated p-value for the correlation of each gene pair. Many tools correlate gene expression data using Pearson correlation. However, the Pearson correlation coefficient assesses significance based on an assumption of normality, while gene expression data does not fit a normal distribution. This motivates our choice of Spearman correlation.

### Identification of bicliques with Apriori

**Figure 3:**
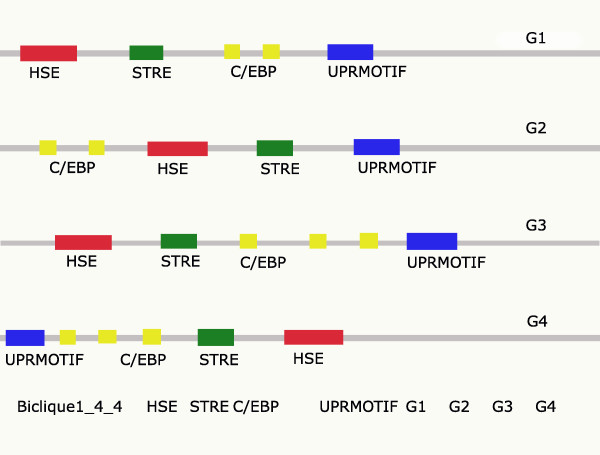
Illustration of a Biclique. Biclique_4_4 is an example of a biclique. Genes Gl, G2, G3, and G4 share regulatory motifs HSE, STRE, C/EBP, and UPRMOTIF on their promoters.

Combinations of *cis*-elements that are significantly over-represented in a geneset are identified using the Apriori data mining algorithm. XcisClique encodes the presence of *cis*-elements in gene promoters with a binary matrix whose rows represent genes and whose columns represent *cis*-elements. The Apriori algorithm finds all maximal sub-matrices of all 1s in this binary matrix [18,19]. A set of cell values is called *maximal *when no more rows can be added without removing columns and vice versa. Each combination of a set of genes and a set of motifs output by the algorithm is called a *biclique *. The *k*^*th *^biclique *I_k _= ⟨G*_*k *_, *M*_*k*_⟩ is defined as a biclique with a set of |*M*_*k*_| motifs, *M*_*k *_in one clique and a set of |*G_k_*| genes, *G*_*k *_in the other. Edges connect members of one clique with all members of the other and are representative of the presence of every motif in *M*_*k *_in every gene in *G*_*k*_. Table 3 illustrates the working of this algorithm with respect to genes and motifs. Figure 3 illustrates the concept of a biclique of genes and patterns, using the MotifSee visualization tool. A biclique does not imply any particular ordered arrangement of patterns. It only indicates the presence of a set of patterns in a set of genes.

### Identification of significant bicliques

The occurrence of a random biclique *M *among all genes of the *Arabidopsis *genome should follow the hypergeometric distribution. A *p*-value is generated for each biclique by calculating the tail probability corresponding to the presence of more than *c *gene promoters with *M *from *n *promoters drawn from the genome set of *N *promoters having C
 MathType@MTEF@5@5@+=feaafiart1ev1aaatCvAUfKttLearuWrP9MDH5MBPbIqV92AaeXatLxBI9gBamrtHrhAL1wy0L2yHvtyaeHbnfgDOvwBHrxAJfwnaebbnrfifHhDYfgasaacH8akY=wiFfYdH8Gipec8Eeeu0xXdbba9frFj0=OqFfea0dXdd9vqai=hGuQ8kuc9pgc9s8qqaq=dirpe0xb9q8qiLsFr0=vr0=vr0dc8meaabaqaciaacaGaaeqabaWaaeGaeaaakeaaimaacqWFce=qaaa@3825@_*M *_promoters with *M *is given by this equation:

ℋtail(N,CM,n,c)=1−∑i=1c(C(n,c)⋅C(N−n,CM−c)C(N,CM))     (1)
 MathType@MTEF@5@5@+=feaafiart1ev1aaatCvAUfKttLearuWrP9MDH5MBPbIqV92AaeXatLxBI9gBamrtHrhAL1wy0L2yHvtyaeHbnfgDOvwBHrxAJfwnaebbnrfifHhDYfgasaacH8akY=wiFfYdH8Gipec8Eeeu0xXdbba9frFj0=OqFfea0dXdd9vqai=hGuQ8kuc9pgc9s8qqaq=dirpe0xb9q8qiLsFr0=vr0=vr0dc8meaabaqaciaacaGaaeqabaWaaeGaeaaakeaaimaacqWFlecsdaWgaaWcbaGaemiDaqNaemyyaeMaemyAaKMaemiBaWgabeaakmaabmaabaGaemOta4KaeiilaWIae8NaXp0aaSbaaSqaaiabd2eanbqabaGccqGGSaalcqWGUbGBcqGGSaalcqWGJbWyaiaawIcacaGLPaaacqGH9aqpcqaIXaqmcqGHsisldaaeWaqaamaabmaabaWaaSaaaeaacqWGdbWqdaqadaqaaiabd6gaUjabcYcaSiabdogaJbGaayjkaiaawMcaaiabgwSixlabdoeadnaabmaabaGaemOta4KaeyOeI0IaemOBa4MaeiilaWIae8NaXp0aaSbaaSqaaiabd2eanbqabaGccqGHsislcqWGJbWyaiaawIcacaGLPaaaaeaacqWGdbWqdaqadaqaaiabd6eaojabcYcaSiab=jq8dnaaBaaaleaacqWGnbqtaeqaaaGccaGLOaGaayzkaaaaaaGaayjkaiaawMcaaaWcbaGaemyAaKMaeyypa0JaeGymaedabaGaem4yamganiabggHiLdGccaWLjaGaaCzcamaabmaabaGaeGymaedacaGLOaGaayzkaaaaaa@7372@

Bicliques from the output of the Apriori algorithm are filtered using *False Discovery Rate (FDR) *[26], applied to the ℋ
 MathType@MTEF@5@5@+=feaafiart1ev1aaatCvAUfKttLearuWrP9MDH5MBPbIqV92AaeXatLxBI9gBamrtHrhAL1wy0L2yHvtyaeHbnfgDOvwBHrxAJfwnaebbnrfifHhDYfgasaacH8akY=wiFfYdH8Gipec8Eeeu0xXdbba9frFj0=OqFfea0dXdd9vqai=hGuQ8kuc9pgc9s8qqaq=dirpe0xb9q8qiLsFr0=vr0=vr0dc8meaabaqaciaacaGaaeqabaWaaeGaeaaakeaaimaacqWFlecsaaa@3763@_*tail*_(*N*, C
 MathType@MTEF@5@5@+=feaafiart1ev1aaatCvAUfKttLearuWrP9MDH5MBPbIqV92AaeXatLxBI9gBamrtHrhAL1wy0L2yHvtyaeHbnfgDOvwBHrxAJfwnaebbnrfifHhDYfgasaacH8akY=wiFfYdH8Gipec8Eeeu0xXdbba9frFj0=OqFfea0dXdd9vqai=hGuQ8kuc9pgc9s8qqaq=dirpe0xb9q8qiLsFr0=vr0=vr0dc8meaabaqaciaacaGaaeqabaWaaeGaeaaakeaaimaacqWFce=qaaa@3825@_*M*_, *n*, *c*) values. The default FDR parameter in XcisClique is 0.05. Ranks are assigned to bicliques in increasing order of their *p-*values.

### Evaluation of genesets using gene expression data

For any geneset *G *of *Arabidopsis *genes, we compute the Spearman correlation coefficients *ρ*(*v*_*g*1_, *v*_*g*2_), as described earlier. Each ρ *(v*_*g*1_, *v*_*g*2 _lies between -1 and 1, with 0 meaning uncorrelated, 1 meaning completely correlated, and -1 meaning completely oppositely correlated. Since a negative correlation may be as biologically significant as a positive correlation, we use the absolute value |ρ *(v*_*g*1_,*v*_*g*2_) to avoid unwanted cancellation of negative and positive correlations. We define the Sum of Absolute Values (SAV) statistic *S(G) *to be the sum of these absolute values, namely:

S(G)=∑g1,g2∈G|ρ(g1,g2)|,     (2)
 MathType@MTEF@5@5@+=feaafiart1ev1aaatCvAUfKttLearuWrP9MDH5MBPbIqV92AaeXatLxBI9gBaebbnrfifHhDYfgasaacH8akY=wiFfYdH8Gipec8Eeeu0xXdbba9frFj0=OqFfea0dXdd9vqai=hGuQ8kuc9pgc9s8qqaq=dirpe0xb9q8qiLsFr0=vr0=vr0dc8meaabaqaciaacaGaaeqabaqabeGadaaakeaacqWGtbWudaqadaqaaiabdEeahbGaayjkaiaawMcaaiabg2da9maaqafabaWaaqWaaeaaiiGacqWFbpGCdaqadaqaaiabdEgaNnaaBaaaleaacqaIXaqmaeqaaOGaeiilaWIaem4zaC2aaSbaaSqaaiabikdaYaqabaaakiaawIcacaGLPaaaaiaawEa7caGLiWoaaSqaaiabdEgaNnaaBaaameaacqaIXaqmaeqaaSGaeiilaWIaem4zaC2aaSbaaWqaaiabikdaYaqabaWccqGHiiIZcqWGhbWraeqaniabggHiLdGccqGGSaalcaWLjaGaaCzcamaabmaabaGaeGOmaidacaGLOaGaayzkaaaaaa@4D16@

where *g*_1 _ranges over all of the genes for which gene expression data are available. The probability of observing a SAV greater than or equal to *S(G) *is given by *p*_*r*_(*S*(*G*)). This is calculated by sampling the distribution of *S(G) *in the genome. Binding a class of transcription factors in response to a set of treatments might induce transcription of some genes in the biclique while repressing other genes in the same biclique. The sum of absolute values of ρ is an indication of how tightly (both negatively and positively) correlated the geneset in a biclique is. Supplementary Figures 2 [see Additional file 2] and 3 [see Additional file 3] illustrate the probability density function and the cumulative distribution function for *S *respectively, for a geneset of size 6.

### Combined p-value for a biclique

The final, combined *p*-value of a biclique *I *= ⟨*G*', *P'*⟩ is the product

ℋ
 MathType@MTEF@5@5@+=feaafiart1ev1aaatCvAUfKttLearuWrP9MDH5MBPbIqV92AaeXatLxBI9gBamrtHrhAL1wy0L2yHvtyaeHbnfgDOvwBHrxAJfwnaebbnrfifHhDYfgasaacH8akY=wiFfYdH8Gipec8Eeeu0xXdbba9frFj0=OqFfea0dXdd9vqai=hGuQ8kuc9pgc9s8qqaq=dirpe0xb9q8qiLsFr0=vr0=vr0dc8meaabaqaciaacaGaaeqabaWaaeGaeaaakeaaimaacqWFlecsaaa@3763@_*tail*_(*N*, C
 MathType@MTEF@5@5@+=feaafiart1ev1aaatCvAUfKttLearuWrP9MDH5MBPbIqV92AaeXatLxBI9gBamrtHrhAL1wy0L2yHvtyaeHbnfgDOvwBHrxAJfwnaebbnrfifHhDYfgasaacH8akY=wiFfYdH8Gipec8Eeeu0xXdbba9frFj0=OqFfea0dXdd9vqai=hGuQ8kuc9pgc9s8qqaq=dirpe0xb9q8qiLsFr0=vr0=vr0dc8meaabaqaciaacaGaaeqabaWaaeGaeaaakeaaimaacqWFce=qaaa@3825@_*M*_, *n*, *c*)·*pr*(*S*(*G*))

of the hypergeometric tail probability from analysis of the biclique *P' *(Equation 1) and the SAV *p-*value from expression analysis of *G' *(Equation 2).

## Results

To evaluate the effectiveness of the XcisClique system, we performed three case studies that applied XcisClique to different genesets and a common set of known regulatory motifs. Case study 1 employs a geneset of 11 AT genes up-regulated by cold stress. Case study 2 employs a geneset of 14 AT genes down-regulated by cold stress. Case study 3 analyzes 113 AT genes involved in senescence.

### Case study 1: metabolism genes up-regulated after cold stress

**Figure 4:**
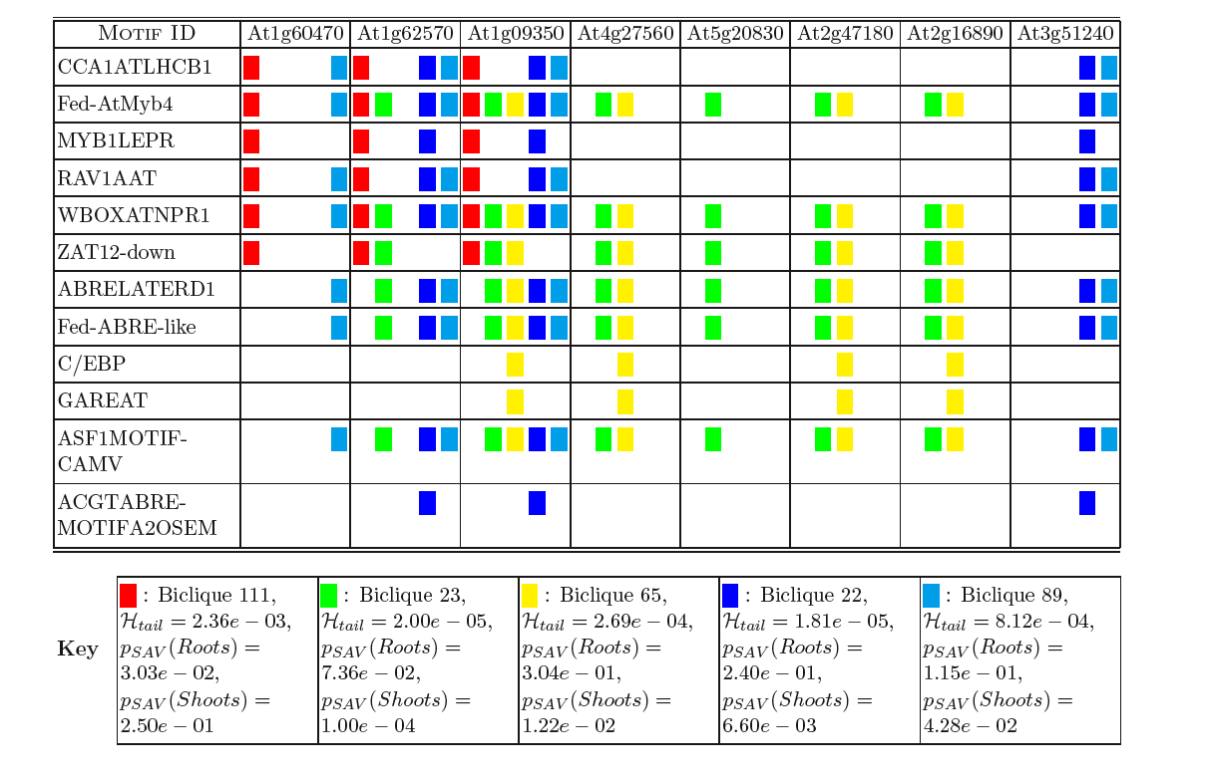
Selected significant motif combinations for Case Study 1. Edges in a biclique are represented by boxes of a particular color. The presence of a biclique box in a motif row *x *and gene column *y *indicates the presence of motif *x *in the promoter of gene *y *. For e.g., all red boxes represent edges in Biclique 111.

For our first case study, we selected a set of 11 AT genes (identified in Supplementary Table 1 [see Additional file 7]) that are involved in carbohydrate metabolism and secondary metabolism and that are up-regulated long-term by cold stress [27]. The Apriori algorithm identified 193 bicliques. After False Discovery Rate (FDR) correction of motifset significance at the 0.05 level, 177 significant bicliques remained. Figure 4 details five of these bicliques that were identified as statistically over-represented both by the hypergeometric (motifset) and SAV (gene expression, see methods) analyses. The motifs in these bicliques include CRT- or DRE-like elements, where the inducible transcription factors CBF1, CBF2, and CBF3 bind [28,29], as well as motifs associated with other abiotic stresses such as water stress (ABRE-like motifs), and, unexpectedly, motifs that have been discovered in pathogen or salicylic acid responsive genes, such as the WBOXATNPR1 [30] and ASF1MOTIFCAMV elements (See the web site for details about these regulatory motifs). The presence of these biotic stress related motifs shows that these genes might play a role not only in abiotic stresses but also in biotic stresses.

Another unexpected motif is CCA1ATLHCB1, the binding site of the Circadian Cycle Associated protein (CCA1), a Myb-related transcription factor [31]. Recent studies on cold-response in AT have shown that CBF transcription factors are regulated by the circadian cycle, with the highest expression observed when plants are transfered to a lower temperature 4 hours after dawn. [32]. The genes analyzed in this group are not CBF transcription factors, but two of the genes that contain the CCA1ATLHCB1 motif (Figure 4) increase their expression within one hour of stress (Supplementary Figure 5 [see Additional file 5] and [27]), gradually increasing until they reach a maximum after 12 hours. Therefore, the initial response of these genes might be due to CCA1 induction and the peak reached by CBF induction.

Biclique 111 (biclique rank in the analysis set found on the web site) is interesting because it contains three genes that are part of the CBF regulon [33] and the motifs contained in this set follow the particular order MYB1LEPR, WBOX, and CCA1ATLHCB1 (Supplementary Figure [see Additional file 4]). Two of the genes that belong to this motifset (Atlg62570 and Atlg60470) are putative galactinol synthase genes. These genes are part of the raffinose biosynthesis pathway, which accumulates in plants treated by cold and drought [34]; raffinose is a sugar that is thought to act as an osmoprotectant under cold and drought.

*Biclique 23 is particularly interesting, because its tight co-regulation is supported not only by the gene expression data but also the results of *[27]*and *[33]*where these genes show a peak up-regulation after 24 hours of cold stress in plate and soil experiments*. Four genes (Atlg09350, Atlg62570, At2gl6890, At5g20830) in this biclique belong to the CBF regulon, and four genes (Atlg09350, Atlg62570, At2gl6890, At4g27180) have the DRECRTCOREAT consensus motif [27,33], which explains their similar expression under cold stress (Supplementary Figure 5 [see Additional file 5]). Biclique 23 also show up-regulation under salt and osmotic stress in shoots. Water stress related motifs Fed-ABRE-like and Fed-AtMyb4 [23] are located within 600 bps of the transcription start site in the upstream regions of these genes, so this particular arrangement of motifs might be responsible for their expression under other comparable stresses (cold, osmotic, and salt) (Supplementary Figure 5 [see Additional file 5]).

### Case study 2: metabolism genes down-regulated after cold stress

The promoters of 14 metabolism genes (identified in Supplementary Table 2 [see Additional file 7]) shown by [27] to be down-regulated after cold stress were analyzed using XcisClique. The Apriori algorithm identified 336 bicliques, which, after a correction with an FDR of 0.05 resulted in 270 significant bicliques. Table 1 shows selected significant motif combinations found for these genes in shoots and roots. [33] have shown that many of the genes that were down-regulated by cold were also down-regulated by over-expression of the CBF or ZAT12 transcription factors. They found putative motifs responsible for down-regulation, but none of the genes that we have studied were shown to be down-regulated by over-expression of CBF and ZAT12 transcription factors [33]. This explains why we did not find significant bicliques containing these motifs in promoters of these genes, and even if they might be present individually, their presence was not associated with a significant motif combination. Genes in biclique 203 show down-regulation under cold stress, but also up-regulation under salt stress (Supplementary Figure 6 [see Additional file 5]), which is a novel observation. This response could be explained by the presence of the combination of ABRELATERD1, and MYCATRD22 which are binding sites of transcription factors responsive to ABA and drought [35], respectively, but have also been found in salt stress induced genes [35].

*A novel heat shock element associated with negative regulation of transcription is included in the motif arrangement in biclique *35 (*Supplementary *Figure 7 [see Additional file 6]). The heat shock element binding site is formed by alternate repeats of the pentamer 5'-nGAAn-3' (5'-nTTCn-3' on the reverse strand). It includes a mutation in the A/T nucleotides of the pentamer [36]. The HSE motif found in these genes shows a mutation in an A/T in the 1st and the 3rd pentamer of the element and therefore, represents a *cis*-element distinct from the sequence of the canonical HSE. These genes also show down-regulation under heat stress (Supplementary Figure 7 [see Additional file 6]). Therefore this mutated HSE motif might be a specific binding site for the class B of heat shock factors, which are negative regulators of transcription [37].

### Case study 3: senescence genes

An input set of 113 senescence responsive genes in AT (identified in Supplementary Table 3 [see Additional file 7]) were analyzed using XcisClique. These genes are taken from [24], and show up-regulation during leaf senescence. These genes are involved in various processes, including protein degradation, oxidation, and detoxification. Expression data for 107 of the 113 genes is available. Promoters of length 1200 for the input geneset were scanned for the set of all AT *cis*-elements. Expression data for the gene set were correlated over a set of 9 treatments (identified as Cold, Heat, Drought, Osmotic, Oxidative, Salt, UVB, Genotoxic, Wounding) in shoots. The complete set of results for this analysis can be viewed at the web site. Table 2 shows a selected set of 2 bicliques that have low *p*-values both from sequence and expression data analysis. Regulation of expression of genes related to senescence involves proteolytic degradation [38]. Biclique 31 contains two proteases (Atlg47128, At4g39090), the ubiquitin-conjugating enzyme 1 (Atlgl4400), and a putative membrane protein (Atlg68820) that by electronic annotation of the GO consortium has putative ubiquitin protein kinase activity, which take part in the degradation process that occurs during senescence. The other genes in biclique 31 encode an ABC transporter (Atlg59870), two putative ethylene synthesis regulators (At2g25450, 2-oxoglutarate-dependent dioxygenase similar to tomato ethylene synthesis regulatory protein E8, and At5gl0860 a CBS domain protein that binds to ATP, ADP, and SAM), and metal binding proteins (At2g26560, patatin like protein with oxidoreductase activity, acting on iron-sulfur proteins as donors, and At3g09390, metallothionein protein). These genes share the ELRECOREPCRP1 motif (Elicitor Responsive Element core of parsley PR1), where WRKY1 transcription factors binds [39,40]. Programmed cell death is observed in plants not only during senescence but also during the hypersensitive response after pathogen attack; therefore, the presence of the ELRECOREPCRP1 motif in these genes suggests up-regulation of these genes after pathogen attack.

Regulation of UV-B responses is associated with specific variations on a consensus sequence. The Fed-HBF or H-box motif has also been shown to be involved in response to oxidative stress (ozone in particular) and/or pathogen attack and is therefore related to cell death [23]. The transcription factor that binds to this *cis*-element belongs to the bZIP transcription family and binds also to a G-box motif [41]. The G-box motif is a palindromic sequence (CACGTG) that is a specific example of the partially defined DPBFCOREDCDC3 consensus sequence (ACACNNG) whose transcription factors also belong to the bZIP family [42]. Genes in biclique 3854 contain the Fed-HBF and DPBFCOREDCDC3 motifs. Four of these genes are involved in proteolysis or protein catabolism (Atlg21670, Atlg47128, Atlg53750, At5g60360). Five genes have precise matches for the G-box motif: Atlg21670, Atlg53750, Atlg78080, At3gl2120, and At5g60360. Four of these genes also show up-regulation under UV-B stress, while genes that match the DPBFCOREDCDC3 motif but do not match the specific G-box motif show down-regulation under UV-B stress (Supplementary Figure 8 [see Additional file 6]).

The H-box and the G-box are also present in the promoter of the chalcone synthase gene (CHS), which catalyzes the first step for the synthesis of flavonoids [43]. CHS is also up-regulated under UV-B stress, since flavonoid molecules can absorb UV-B radiation [44]. Atlg21670 and Atlg53750 are related to protein degradation, while Atlg78080 is a transcription factor (RAP2.4) and At3gl2120 is a fatty acid desaturase (FAD2). These genes are not related to flavonoid synthesis but they might protect the plant against UV-B stress in other ways such as catabolism of damaged proteins by UV-B (Atlg21670, Atlg53750) or signaling/activation of other protective pathways (At3gl2120/Atlg53750).

## Discussion

Several programs have been developed for discovering *cis*-regulatory modules in yeast. The transcriptional mechanisms in yeast are somewhat understood, and there is enough biological data about yeast upon which to base computational findings on. In a higher eukaryote such as *Arabidopsis*, the gene-abundance is much higher (approximately 28,000). While there are databases that identify all TFBSs in yeast, not all TFBSs in AT are known and documented. Only a fraction of TFBSs in AT have documented consensus sequences. Position weight matrices are even rarer with TRANSFAC containing 10 position weight matrices for binding sites in AT. The lack of sufficient biological data in the case of AT makes the validation of promoter discovery tools problematic.

XcisClique provides a novel platform for investigating regulatory motifs in *Arabidopsis *via an integrated infrastructure combining annotated genome data, annotated *cis*-element data, and gene expression data. XcisClique identifies statistically overrepresented bicliques and evaluates each biclique with respect to gene expression data. This gives an indication of the importance of co-occurrence of a set of regulatory elements in a geneset with respect to transcriptional response. The *p*-value of each biclique is a determinant of biological significance as well. To measure the tightness of correlation of genes in a biclique, we needed a statistic. Initially, we considered the simple sum of the Spearman correlation coefficients of all pairs of genes in a biclique, but negative correlations balanced the positive correlations and the simple sum was not a good indicator of how tightly correlated genes in a biclique were. For instance, a set of correlations {0.1, -0.1,0.3} yields the same statistic as the set {0.8, -0.8,0.3}. Obviously, the latter set of genes is more tightly correlated. The sum of absolute values statistic considers the individual contributions of all correlations and is a sharper test of the tightness of correlations in a biclique. Hence, we used this statistic to measure the co-expression of a biclique.

Most transcription factors families in plants are large, therefore there is a possibility that some of their members might be activators, and others, repressors. Since, in most cases, the specific *cis*-elements regions for each of the members of a given transcription factor family have not yet been determined, what is currently available is a consensus sequence serving as a motif rather than a specific sequence. Application of SAV results in the grouping of genes that share a set of these, often partially defined, motifs, with the result that some gene groups that share consensus motifs might be down-regulated compared with other groups under the same experimental conditions. In these cases, we have found that the actual sequence of the motifs in the down-regulated gene group is different from those in the up-regulated group; for example in the case of the DPBFCOREDCDC3 motif in the analysis of the senescence genes that we made (Case study 3). The DPBFCOREDCDC3 consensus sequence (ACACNNG) also subsumes the defined G-box motif sequence (CACGTG). Genes in biclique 3854 contain the DPBFCOREDCDC3 motif. Five genes have matches for the DPBFCOREDCDC3 motif that also correspond to the G-box motif: Atlg21670, Atlg53750, Atlg78080, At3gl2120, and At5g60360. Four of these genes show up-regulation under UV-B stress, while genes that have matches to the DPBFCOREDCDC3 motif but do not to the G-box motif show down-regulation under UV-B stress (Supplementary Figure 8 [see Additional file 6]). The different response/regulation of genes in the biclique can be explained by the different sequences of matches, all of which match the regular expression for the DPBFCOREDCDC3 motif.

Another example is the heat shock factor (HSF) family. Class B HSFs inhibit transcription (Czarneka-Verner et al 2004). These HSFs do not bind to the canonical heat shock element (alternate repeats of 5' -nGAAn- 3'), and therefore, Class B HSFs must bind to another *cis*-sequence in target genes. Our study allowed us to identify *cis*-sequences that are possible candidates for binding of this class of HSFs. The HSE motif in genes of Biclique 35 (Case Study 2) show a mutation in an A/T in the 1st and the 3rd pentamer of the element. These genes also show down-regulation under heat stress (Supplementary Figure 7 [see Additional file 5]). Therefore this HSE motif might be a specific binding site for class B heat shock factors. XcisClique uses only known AT motifs curated from various sources. This ensures that the search space for patterns is limited, not confined by motif lengths, and consists of well-defined, annotated motifs. The biologist has the choice of selecting a subset of relevant motifs, and this makes one of the three inputs (*cis*-elements) biologically directed. The second and third inputs which are the genes being analyzed and the treatment sets over which expression data is to be considered, respectively, are also specified by the biologist. The integration of biological knowledge within XcisClique greatly reduces the final search space and yields more biologically relevant results. XcisClique is scalable to more numerous motifs and treatments. The system has been verified with biological data from *Arabidopsis *. Given adequate sequence and gene expression data, the system is sufficiently generic to accommodate any organism. MotifSee, a visualization component of XcisClique, supports viewing combinations of motifs in gene promoters. A viewer for visualizing gene expression patterns of a set of genes is also integrated into the system.

## Conclusion

Using both motifset significance, assessed using the hypergeometric distribution, and gene expression correlation, assessed using the SAV statistic, ensures that the biological context is present in the final significance value calculated. Consider a set of genes such that every gene in the set is highly correlated to every other gene. The set can be expanded by correlating each gene in the set to every gene in the AT genome. Only those genes are added to the original set, whose correlation coefficient with one of the members of the original set is above a given threshold. This process is not available with the XcisClique web-interface. The enriched set can be input to XcisClique to produce more significant bicliques. Also, conserved arrangements of motifs were observed in significant bicliques. A formalization of the process to identify conserved arrangements is one of the future directions we are pursuing.

## Availability and requirements

The web-based interface for XcisClique is available at . The source code for XcisClique is freely available under the GNU Public License at the following location:  .

The following software components are required to install and run the command line version of XcisClique.

1. Perl 5.8.5 or higher.

2. Perl Modules.

(a) LWP::Simple This Perl module provides a simple, procedural interface to LWP, which is the World-Wide Web library for Perl, a set of Perl modules which provides a sample and consistent application programming interface (API) to the World-Wide Web. *CPAN*

(b) Shell Perl module to run shell commands transparently within Perl. *CPAN*

(c) DBI Perl module for database access. It defines a set of methods, variables, and conventions that provide a consistent database interface, independent of the actual database being used. *CPAN*

(d) DBD::Pg This is the PostgreSQL database driver for the DBI module. *CPAN*

(e) Test::Simple Pre-requisite for DBD::Pg.* CPAN*

(f) Time::localtime Perl module with interfaces to Perl's built-in localtime() function. *CPAN*

(g) Math::Matrix Perl module with functions for multiplication, inversion, and other common matrix operations. *CPAN*

(h) Statistics::Distributions Perl module for calculating critical values and upper probabilities of common statistical distributions such as the Normal distribution, the χ^2 ^distribution, the t distribution, and the F distribution. *CPAN*

(i) PDF Perl module with functions for calculating critical values and probabilities of various statistical distributions, such as the Binomial distribution, the Hypergeometric distribution, and the Gaussian distribution. *Packaged with XcisClique*

(j) Vector Perl module for common vector operations and calculation of Pearson and Spearman correlation coefficients between vectors. *Packaged with XcisClique*

(k) Utilities Perl module with common text processing utility functions such as removing white space from a string. *Packaged with XcisClique*

3. PostgreSQL 7.4.7 or higher.

4. MATLAB 7.0.4 with Statistics toolbox.

## Authors' contributions

AP and LSH conceived of the study, participated in its design and implementation, contributed to the choice of case studies, and drafted the manuscript. The source code for XcisClique has been developed by AP. TMM participated in the design of the system and provided the implementation of the Apriori algorithm. CVR and RG conceived of the biological case studies, ensured biological validity of all methods used in this work, and tested the system.

## Supplementary Material

Additional File 1 Supplementary Figure 1 : This figure illustrates the distribution ρ value for Spearman correlations of the rd29a gene expression vector with all genes of the AT genome. Click here for file

Additional File 2 Supplementary Figure 2 : This figure illustrates the probability density function of the SAV statistic for a geneset of size 6. Click here for file

Additional File 3 Supplementary Figure 3 : This figure illustrates the cumulative distribution function of the SAV statistic for a geneset of size 6. Click here for file

Additional File 4 Supplementary Figure 4 : This figure illustrates motif arrangements in the biclique ranked 111 in analysis 5. Click here for file

Additional File 5 Supplementary Figures 5 and 6 : This is a set of two figures illustrating expression vectors for genes in biclique 23 in Case study 1 and biclique 203 in Case study 2. Click here for file

Additional File 6 Supplementary Figures 7 and 8 : This is a set of two figures illustrating the expression vectors for genes in biclique 35 in Case study 2 and biclique 3854 in Case study 3. Click here for file

Additional File 7 Supplementary Tables : Supplementary tables 1, 2, and 3 list the input genes for Case Studies 1,2, and 3 respectively. Click here for file

## Figures and Tables

**Table 1:** Selected significant motif combinations from Cold down-regulated metabolism genes in shoots

**Biclique**	**Motifset**	**Geneset**	***p- *value**
Biclique 203	ABRELATERD1, ASF1MOTIFCAMV, C/EBP, Fed-SA-induced, GT1CONSENSUS, IBOX, MYBCORE, MYCATERD1, MYCATRD22, MYCCONSENSUSAT, RAV1AAT, WBOXATNPR1	At1g55920, At4g15550, At5g24160	ℋ MathType@MTEF@5@5@+=feaafiart1ev1aaatCvAUfKttLearuWrP9MDH5MBPbIqV92AaeXatLxBI9gBamrtHrhAL1wy0L2yHvtyaeHbnfgDOvwBHrxAJfwnaebbnrfifHhDYfgasaacH8akY=wiFfYdH8Gipec8Eeeu0xXdbba9frFj0=OqFfea0dXdd9vqai=hGuQ8kuc9pgc9s8qqaq=dirpe0xb9q8qiLsFr0=vr0=vr0dc8meaabaqaciaacaGaaeqabaWaaeGaeaaakeaaimaacqWFlecsaaa@3763@_*tail*_*= 5.l7e – 03*,*pSAV(Roots) = *3.86e – 02, *pSAV (Shoots) = *6.60e – 03
Biclique 35	ABRELATERD1, ASF1MOTIFCAMV, C/EBP, Fed-AtMyb4, GAREAT, GT1CONSENSUS HSE2-mut, IBOX, MYB1AT, MYB2CONSENSUSAT, MYBCORE, MYCCONSENSUSAT, RAV1AAT, TBOXATGAPB, WBOXATNPR1	At1gl6410,At3g01500, At5g14740,At5g18170	ℋ MathType@MTEF@5@5@+=feaafiart1ev1aaatCvAUfKttLearuWrP9MDH5MBPbIqV92AaeXatLxBI9gBamrtHrhAL1wy0L2yHvtyaeHbnfgDOvwBHrxAJfwnaebbnrfifHhDYfgasaacH8akY=wiFfYdH8Gipec8Eeeu0xXdbba9frFj0=OqFfea0dXdd9vqai=hGuQ8kuc9pgc9s8qqaq=dirpe0xb9q8qiLsFr0=vr0=vr0dc8meaabaqaciaacaGaaeqabaWaaeGaeaaakeaaimaacqWFlecsaaa@3763@_*tail*_= 1.58e – 05, *pSAV(Roots) = *1.15e – 01,*p*_*sav*_*(Shoots) = *4.28e – 02

**Table 2:** Selected significant motif combinations from AT genes up-regulated during senescence

**Biclique**	**Motifset**	**Geneset**	***p- *value**
Biclique 31	ELRECOREPCRP1, EVENINGAT, Fed-AtMyb4, GT1CONSENSUS, IBOX, MYCCONSENSUSAT, RAV1AAT, WBOXATNPR1	At1g14400, At1g47128, At1g59870, At1g68820, At2g25450, At2g26560, At3g09390, At4g39090, At5g10860	ℋ MathType@MTEF@5@5@+=feaafiart1ev1aaatCvAUfKttLearuWrP9MDH5MBPbIqV92AaeXatLxBI9gBamrtHrhAL1wy0L2yHvtyaeHbnfgDOvwBHrxAJfwnaebbnrfifHhDYfgasaacH8akY=wiFfYdH8Gipec8Eeeu0xXdbba9frFj0=OqFfea0dXdd9vqai=hGuQ8kuc9pgc9s8qqaq=dirpe0xb9q8qiLsFr0=vr0=vr0dc8meaabaqaciaacaGaaeqabaWaaeGaeaaakeaaimaacqWFlecsaaa@3763@_*tail *_= 4.30e – 06, *pSAV(Shoots)*= 8.70e-04
Biclique 3854	DPBFCOREDCDC3, Fed-AtMyb4, Fed-HBF, GT1CONSENSUS, MYCCONSENSUSAT	At1g21670, At1g47128, At1g53750, At1g78080, At3g12120, At4g27020, At5g60360	ℋ MathType@MTEF@5@5@+=feaafiart1ev1aaatCvAUfKttLearuWrP9MDH5MBPbIqV92AaeXatLxBI9gBamrtHrhAL1wy0L2yHvtyaeHbnfgDOvwBHrxAJfwnaebbnrfifHhDYfgasaacH8akY=wiFfYdH8Gipec8Eeeu0xXdbba9frFj0=OqFfea0dXdd9vqai=hGuQ8kuc9pgc9s8qqaq=dirpe0xb9q8qiLsFr0=vr0=vr0dc8meaabaqaciaacaGaaeqabaWaaeGaeaaakeaaimaacqWFlecsaaa@3763@_*tail*_= 4.88e – 02, *psav(Shoots)*= 3.54e-02

**Table 3:** Binary matrix representing the presence of motifs in gene promoters.*G*_1 _through *G*_8 _are hypothetical genes and *M*_1 _through *M*_5 _are hypothetical motifs. A 1 in cell (*i*, *j*) indicates the presence of motif *M*_*j *_in the promoter of gene *G*_*i*_. A 0 indicates a corresponding absence.

	M_1_	M_2_	M_3_	M_4_	M_5_
G1	1	1	0	0	0
G2	1	1	1	1	1
G3	0	0	0	0	0
G4	0	0	1	1	1
G5	0	0	0	0	0
G6	0	0	1	1	1
G7	0	0	0	0	0
G8	1	1	1	1	1

Bicliques

⟨*M*_1_,*M*_2_,*G*_1_,*G*_2_,*G*_8_⟩

⟨*M*_3_, *M*_4_, *M*_5_, *G*_2_, *G*_4_, *G*_6_, *G*_8 _⟩

⟨*M*_1_, *M*_2_, *M*_3_, *M*_4_, *M*_5_, *G*_2_, *G*_8 _⟩
