# Supplementary material for: XcisClique: analysis of regulatory bicliques
Source: BMC Bioinformatics. 2006 Apr 21;7:218. doi: 10.1186/1471-2105-7-218 (PMC1513260; doi:10.1186/1471-2105-7-218)
Supplement: Additional File 1 — Supplementary Figure 1 : This figure illustrates the distribution ρ value for Spearman correlations of the rd29a gene expression vector with all genes of the AT genome. [file 1471-2105-7-218-S1.pdf]

Supplementary Figures

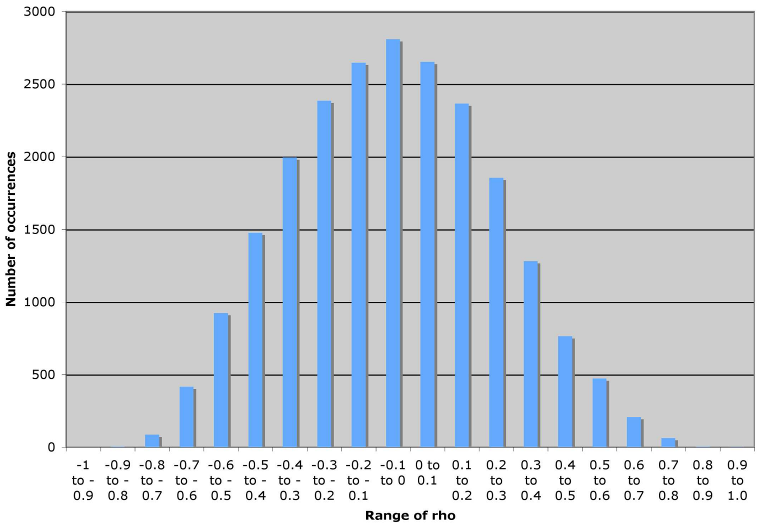

Supplementary Figure 1: Distribution of the ρ value for correlations of the rd29a gene expression vector with all genes of the AT genome.
