# Supplementary figures and images for: XcisClique: analysis of regulatory bicliques
Source: BMC Bioinformatics. 2006 Apr 21;7:218. doi: 10.1186/1471-2105-7-218 (PMC1513260; doi:10.1186/1471-2105-7-218)

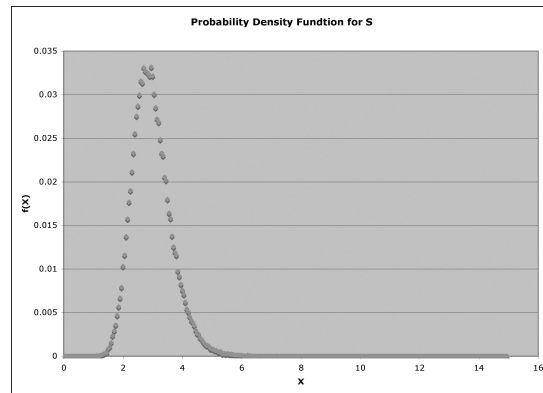

Supplementary Figure 2: Distribution of  $S$  for sample size 6: Probability Density Function

Supplement: Additional File 2 — Supplementary Figure 2 : This figure illustrates the probability density function of the SAV statistic for a geneset of size 6. [file 1471-2105-7-218-S2.pdf]

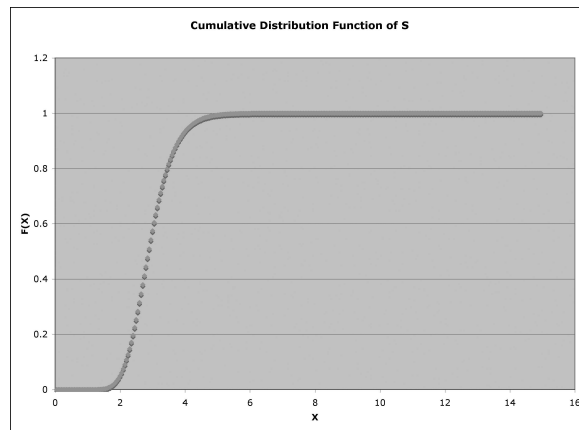

Supplementary Figure 3: Distribution of  $S$  for sample size 6: Cumulative Distribution Function

Supplement: Additional File 3 — Supplementary Figure 3 : This figure illustrates the cumulative distribution function of the SAV statistic for a geneset of size 6. [file 1471-2105-7-218-S3.pdf]

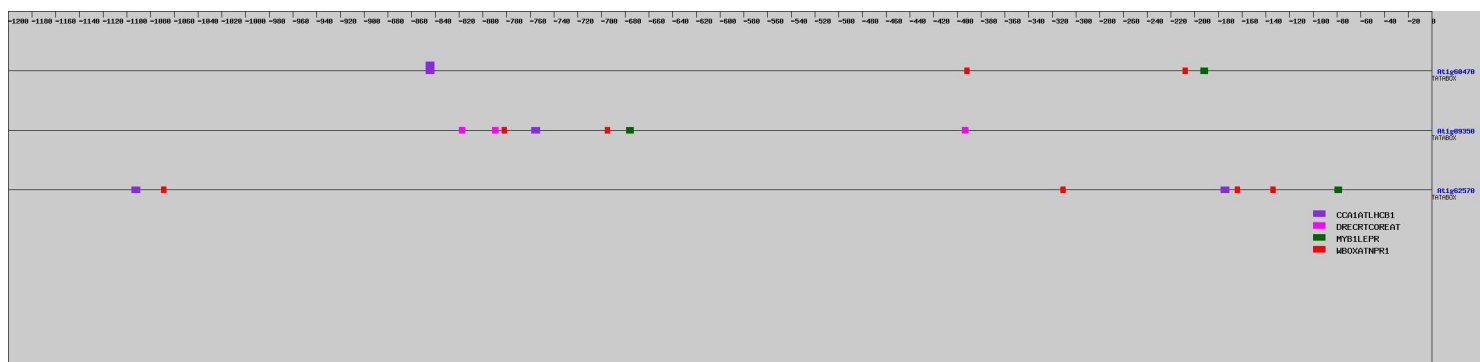

Supplementary Figure 4: Motif Arrangements in Biclique ranked 111 in analysis 5

Supplement: Additional File 4 — Supplementary Figure 4 : This figure illustrates motif arrangements in the biclique ranked 111 in analysis 5. [file 1471-2105-7-218-S4.pdf]
