# Supplementary material for: XcisClique: analysis of regulatory bicliques
Source: BMC Bioinformatics. 2006 Apr 21;7:218. doi: 10.1186/1471-2105-7-218 (PMC1513260; doi:10.1186/1471-2105-7-218)
Supplement: Additional File 5 — Supplementary Figures 5 and 6 : This is a set of two figures illustrating expression vectors for genes in biclique 23 in Case study 1 and biclique 203 in Case study 2. [file 1471-2105-7-218-S5.pdf]

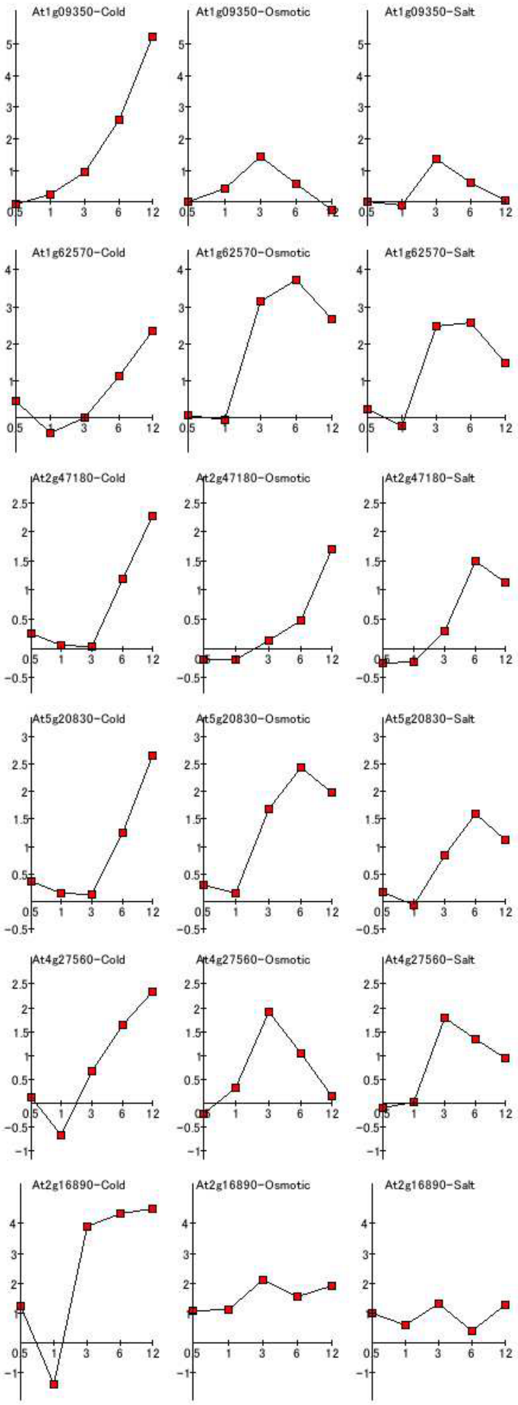

Supplementary Figure 5: Expression vectors for genes in Biclique 23

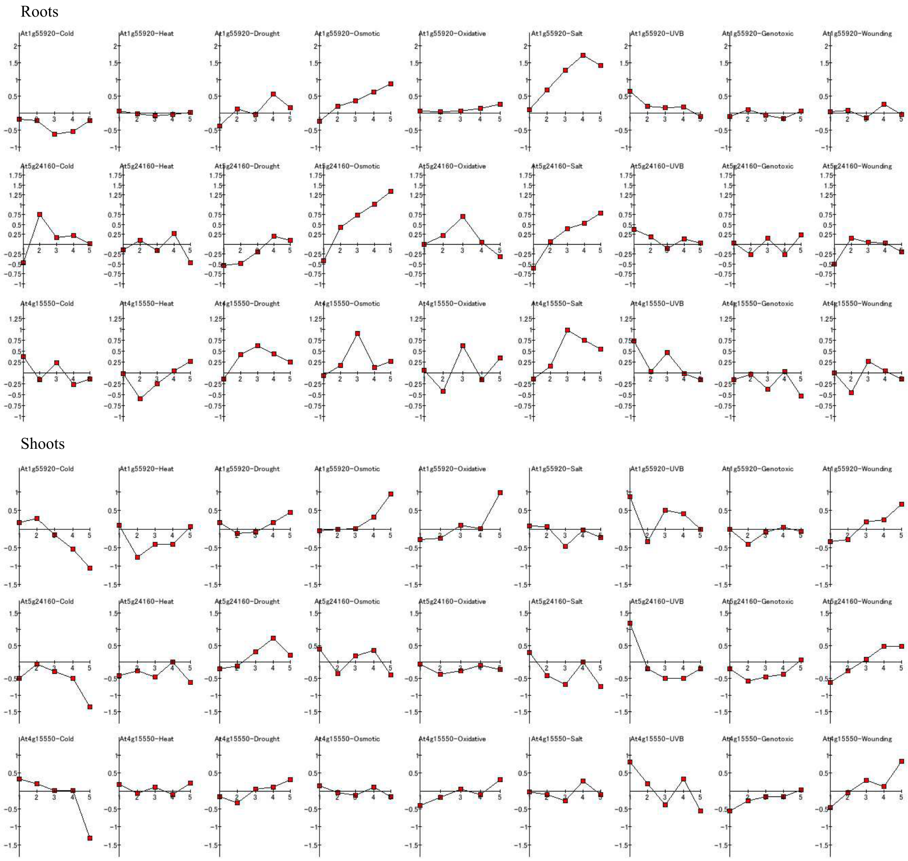

Supplementary Figure 6: Expression vectors for genes in Biclique 203
