# Supplementary material for: XcisClique: analysis of regulatory bicliques
Source: BMC Bioinformatics. 2006 Apr 21;7:218. doi: 10.1186/1471-2105-7-218 (PMC1513260; doi:10.1186/1471-2105-7-218)
Supplement: Additional File 6 — Supplementary Figures 7 and 8 : This is a set of two figures illustrating the expression vectors for genes in biclique 35 in Case study 2 and biclique 3854 in Case study 3. [file 1471-2105-7-218-S6.pdf]

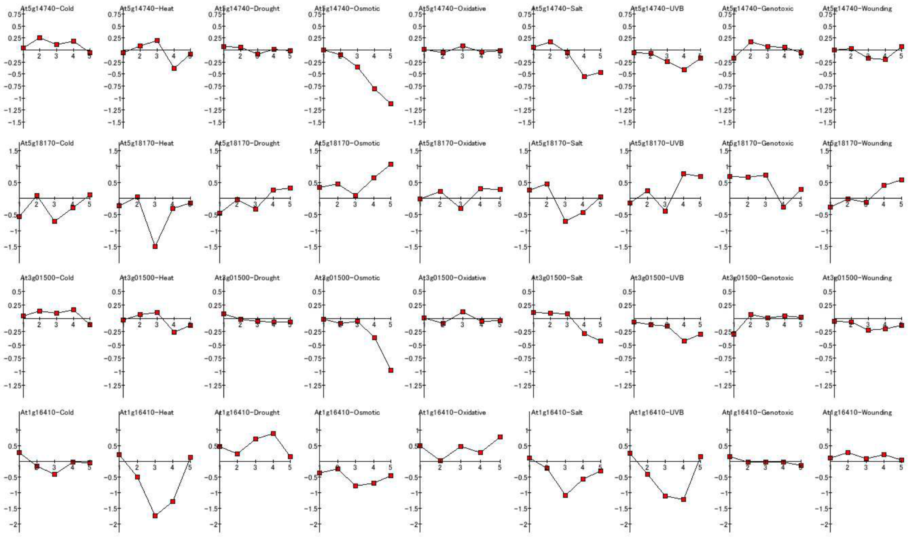

Supplementary Figure 7: Expression vectors for genes in Biclique 35

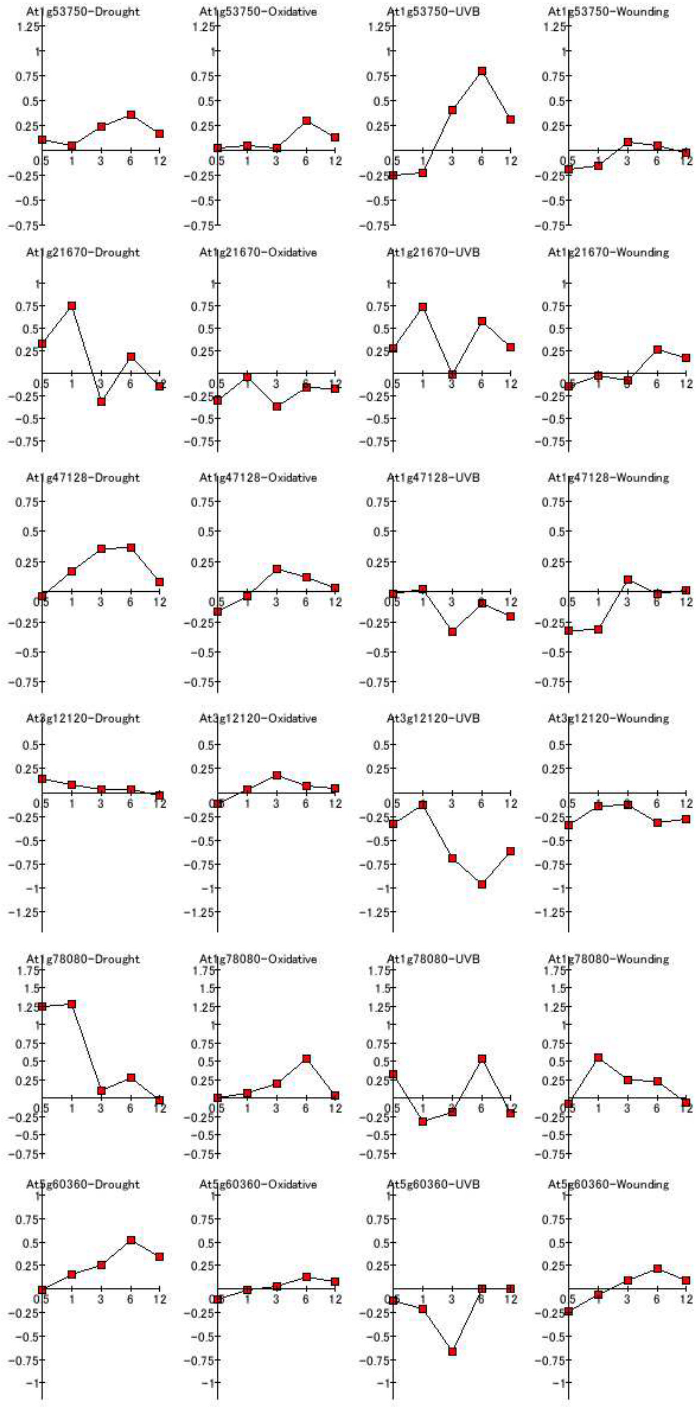

Supplementary Figure 8: Expression vectors for genes in Biclique 3854
