# Supplementary material for: XcisClique: analysis of regulatory bicliques
Source: BMC Bioinformatics. 2006 Apr 21;7:218. doi: 10.1186/1471-2105-7-218 (PMC1513260; doi:10.1186/1471-2105-7-218)
Supplement: Additional File 7 — Supplementary Tables : Supplementary tables 1, 2, and 3 list the input genes for Case Studies 1,2, and 3 respectively. [file 1471-2105-7-218-S7.pdf]

Supplementary Tables

Supplementary Table 1: The 11 genes in the geneset for Case Study 1

Gene ID	Annotation
At1g62570	flavin-containing monooxygenase family protein / FMO family protein
At1g09350	galactinol synthase, putative
At1g60470	galactinol synthase, putative
At2g47180	galactinol synthase, putative
At4g17090	beta-amylase (CT-BMY) / 1,4-alpha-D-glucan maltohydrolase
At5g20830	sucrose synthase / sucrose-UDP glucosyltransferase (SUS1)
At2g16890	UDP-glucuronosyl/UDP-glucosyl transferase family protein
At3g51240	naringenin 3-dioxygenase / flavanone 3-hydroxylase (F3H)
At3g55120	chalcone-flavanone isomerase / chalcone isomerase (CHI)
At4g27560	glycosyltransferase family protein
At5g08640	flavonol synthase 1 (FLS1)

Supplementary Table 2: The 14 genes in the geneset for Case Study 2

Gene ID	Annotation
At5g18170	glutamate dehydrogenase 1 (GDH1)
At1g28670	lipase
At2g35690	acyl-CoA oxidase, putative
At1g55920	serine O-acetyltransferase, putative
At4g22690	cytochrome P450 family protein
At1g10360	glutathione S-transferase, putative
At1g16410	cytochrome P450, putative
At2g22330	cytochrome P450, putative
At2g23600	hydrolase, alpha/beta fold family protein
At2g29340	short-chain dehydrogenase/reductase (SDR) family protein
At4g15550	UDP-glucose:indole-3-acetate beta-D-glucosyltransferase (IAGLU)
At5g24160	squalene monooxygenase 1,2 / squalene epoxidase 1,2 (SQP1,2)
At5g14740	carbonic anhydrase 2 / carbonate dehydratase 2 (CA2) (CA18)
At3g01500	carbonic anhydrase 1, chloroplast / carbonate dehydratase 1 (CA1)

Supplementary Table 3: The 113 senescent-responsive genes in the geneset for Case Study 3

Gene ID	Annotation
At1g07600	metallothionein-like protein 1A (MT-1A) (MT-Q) (MT-2)
At1g09500	cinnamyl-alcohol dehydrogenase family / CAD family
At1g13990	expressed protein
At1g14400	ubiquitin-conjugating enzyme 1 (UBC1)
At1g18210	calcium-binding protein, putative
At1g19570	dehydroascorbate reductase, putative
At1g20620	catalase 3 (SEN2)
At1g21670	expressed protein
At1g30420	ATP-binding cassette transport protein, putative
At1g30460	zinc finger (CCCH-type) family protein / YT521-B-like family protein
At1g35160	14-3-3 protein GF14 phi (GRF4)
At1g47128	cysteine proteinase (RD21A) / thiol protease
At1g51200	zinc finger (AN1-like) family protein
At1g52040	jacalin lectin family protein
At1g52880	no apical meristem (NAM) family protein
At1g53580	hydroxyacylglutathione hydrolase, putative / glyoxalase II, putative
At1g53750	26S proteasome AAA-ATPase subunit (RPT1a)
At1g58360	amino acid permease I (AAP1)
At1g59870	ABC transporter family protein
At1g63010	SPX (SYG1/Pho81/XPR1) domain-containing protein
At1g67840	ATP-binding region, ATPase-like domain-containing protein
At1g68820	membrane protein, putative
At1g70900	expressed protein
At1g71950	expressed protein
At1g73260	trypsin and protease inhibitor family protein / Kunitz family protein
At1g73325	trypsin and protease inhibitor family protein / Kunitz family protein
At1g73750	expressed protein

Continued on next page

Supplementary Table 3: Continued

Gene ID	Annotation
At1g75280	isoflavone reductase, putative
At1g75380	wound-responsive protein-related
At1g78080	AP2 domain-containing transcription factor RAP2.4
At1g78380	glutathione S-transferase, putative
At2g05540	glycine-rich protein
At2g20860	lipoic acid synthase (LIP1)
At2g21660	glycine-rich RNA-binding protein (GRP7)
At2g21950	SKP1 interacting partner 6 (SKIP6)
At2g22240	inositol-3-phosphate synthase isozyme 2 / myo-inositol-1-phosphate synthase 2 / MI-1-P synthase 2 / IPS 2
At2g23980	cyclic nucleotide-regulated ion channel / cyclic nucleotide-gated channel (CNGC6)
At2g25450	2-oxoglutarate-dependent dioxygenase, putative
At2g26560	patatin, putative
At2g39900	LIM domain-containing protein
At2g42690	lipase, putative
At2g43290	calmodulin-like protein (MSS3)
At2g43770	transducin family protein / WD-40 repeat family protein
At2g45210	auxin-responsive protein-related
At3g02040	glycerophosphoryl diester phosphodiesterase family protein
At3g03720	amino acid permease family protein
At3g07590	small nuclear ribonucleoprotein D1, putative / snRNP core protein D1, putative / Sm protein D1, putative
At3g09390	metallothionein protein, putative (MT2A)
At3g12120	omega-6 fatty acid desaturase, endoplasmic reticulum (FAD2) / delta-12 desaturase
At3g15580	autophagy 8i (APG8i)
At3g15730	phospholipase D alpha 1 / PLD alpha 1 (PLDALPHA1) (PLD1) / choline phosphatase 1
At3g16640	translationally controlled tumor family protein

Continued on next page

Supplementary Table 3: Continued

Gene ID	Annotation
At3g17790	acid phosphatase type 5 (ACP5)
At3g22600	protease inhibitor/seed storage/lipid transfer protein (LTP) family protein
At3g23920	beta-amylase, putative / 1,4-alpha-D-glucan maltohydrolase, putative
At3g25480	rhodanese-like domain-containing protein
At3g25760	early-responsive to dehydration stress protein (ERD12)
At3g26100	regulator of chromosome condensation (RCC1) family protein
At3g44100	MD-2-related lipid recognition domain-containing protein / ML domain-containing protein
At3g44300	nitrilase 2 (NIT2)
At3g44720	prephenate dehydratase family protein
At3g46000	actin-depolymerizing factor, putative (ADF2)
At3g48000	aldehyde dehydrogenase (ALDH2)
At3g51130	expressed protein
At3g51730	saposin B domain-containing protein
At3g52800	zinc finger (AN1-like) family protein
At3g55430	glycosyl hydrolase family 17 protein / beta-1,3-glucanase, putative
At3g62550	universal stress protein (USP) family protein
At4g01610	cathepsin B-like cysteine protease, putative
At4g03280	cytochrome B6-F complex iron-sulfur subunit, chloroplast / Rieske iron-sulfur protein / plastoquinol-plastocyanin reductase (petC)
At4g11360	zinc finger (C3HC4-type RING finger) family protein (RHA1b)
At4g11600	glutathione peroxidase, putative
At4g11910	expressed protein
At4g12290	copper amine oxidase, putative
At4g13250	short-chain dehydrogenase/reductase (SDR) family protein
At4g16190	cysteine proteinase, putative
At4g16740	terpene synthase/cyclase family protein
At4g18280	glycine-rich cell wall protein-related

Continued on next page

Supplementary Table 3: Continued

Gene ID	Annotation
At4g24220	expressed protein
At4g27020	expressed protein
At4g27830	glycosyl hydrolase family 1 protein
At4g30210	NADPH-cytochrome p450 reductase, putative / NADPH-ferrihemoprotein reductase, putative
At4g30270	MERI-5 protein (MERI-5) (MERI5B) / endo-xyloglucan transferase / xyloglucan endo-1,4-beta-D-glucanase (SEN4)
At4g30440	NAD-dependent epimerase/dehydratase family protein
At4g32940	vacuolar processing enzyme gamma / gamma-VPE
At4g35750	Rho-GTPase-activating protein-related
At4g37390	auxin-responsive GH3 family protein
At4g37990	mannitol dehydrogenase, putative (ELI3-2)
At4g39090	cysteine proteinase RD19a (RD19A) / thiol protease
At4g39670	expressed protein
At5g01600	ferritin 1 (FER1)
At5g02380	metallothionein protein 2B (MT-2B)
At5g08410	ferredoxin-thioredoxin reductase, putative
At5g10650	zinc finger (C3HC4-type RING finger) family protein
At5g10860	CBS domain-containing protein
At5g11520	aspartate aminotransferase, chloroplast / transaminase A (ASP3) (YLS4)
At5g13170	nodulin MtN3 family protein
At5g17220	glutathione S-transferase, putative
At5g18100	superoxide dismutase [Cu-Zn] / copper/zinc superoxide dismutase (CSD3)
At5g19540	expressed protein
At5g24160	squalene monooxygenase 1,2 / squalene epoxidase 1,2 (SQP1,2)
At5g24770	vegetative storage protein 2 (VSP2)
At5g24780	vegetative storage protein 1 (VSP1)
At5g34850	calcineurin-like phosphoesterase family protein

Continued on next page

Supplementary Table 3: Continued

Gene ID	Annotation
At5g43850	acireductone dioxygenase (ARD/ARD') family protein
At5g48380	leucine-rich repeat family protein / protein kinase family protein
At5g53350	ATP-dependent Clp protease ATP-binding subunit ClpX1 (CLPX)
At5g53730	harpin-induced family protein / HIN1 family protein / harpin-responsive family protein
At5g54250	cyclic nucleotide-regulated ion channel / cyclic nucleotide-gated channel (CNGC4)
At5g57655	xylose isomerase family protein
At5g59310	lipid transfer protein 4 (LTP4)
At5g60360	cysteine proteinase, putative / AALP protein (AALP)
At1g05340	expressed protein

Concluded
